# Tartrazine Modifies the Activity of *DNMT* and *HDAC* Genes—Is This a Link between Cancer and Neurological Disorders?

**DOI:** 10.3390/nu15132946

**Published:** 2023-06-28

**Authors:** Afshin Zand, Sodbuyan Enkhbilguun, John M. Macharia, Ferenc Budán, Zoltán Gyöngyi, Timea Varjas

**Affiliations:** 1 Department of Public Health Medicine, Medical School, University of Pécs, H-7624 Pécs, Hungary; af.zand@gmail.com (A.Z.); enkhbilguunsod@gmail.com (S.E.); vtimi_68@yahoo.com (T.V.); 2 Doctoral School of Health Sciences, Faculty of Health Science, University of Pécs, H-7621 Pécs, Hungary; johnmacharia@rocketmail.com; 3 Institute of Transdisciplinary Discoveries, Medical School, University of Pécs, H-7624 Pécs, Hungary; budfer2@gmail.com; 4 Institute of Physiology, Medical School, University of Pécs, H-7624 Pécs, Hungary

**Keywords:** *DNMT*, *HDAC*, tartrazine, additive, mice, qRT-PCR, gene expression, azo-dye, food colorant, epigenetics

## Abstract

In recent years, artificial additives, especially synthetic food colorants, were found to demonstrate wider properties compared to their natural equivalents; however, their health impact is still not totally mapped. Our study aimed to determine the long-term (30 and 90 days) exposure effect of one of the commonly used artificial food colorants, tartrazine, on NMRI mice. The applied dose of tartrazine referred to the human equivalent dose for acceptable daily intake (ADI). Further, we evaluated its impact on the transcription of a range of epigenetic effectors, members of the DNA methyltransferase (*DNMT*) as well as histone deacetylase (*HDAC)* families. Following the exposure, organ biopsies were collected from the lungs, kidneys, liver, and spleen, and the gene expression levels were determined by real-time quantitative reverse transcription PCR (RT-qPCR). Our results demonstrated significant upregulation of genes in the tested organs in various patterns followed by the intake of tartrazine on ADI. Since *DNMT* and *HDAC* genes are involved in different steps of carcinogenesis, have roles in the development of neurological disorders and the effect of dose of everyday exposure is rarely studied, further investigation is warranted to study these possible associations.

## 1. Introduction

Color additives have been widely used since 1500 BC and currently are extensively used by food manufacturers as a vital criterion for food choice. Among them, synthetic food colorants are more favorable due to their stability, low cost, and coloring properties. These artificial food colorants are available in different varieties and colors [1]. Often, we consume them unknowingly. Therefore, it is essential to study the biological consequences of food colorant usage [2].

The consumption of artificial food colorants below the acceptable daily intake (ADI) does not cause any harm, but it may cause for example many disorders among children due to their low body weight [3]. In developing countries, children are the major consumers of artificial food colorants, which is the reason they are at high risk of several illnesses such as asthma [4]. The total world colorant production is estimated to be 80,000,000 tons per year [5].

Azo dyes are a group of vivid synthetic food colorants including tartrazine (TRZ; trisodium-5-hydroxy-1-(4-sulfonatophenyl)-4-(4-sulfonatophenyl-azo)-H-pyrazol-3-carboxylate) and represent about two-thirds of all synthetic dyes, which are undoubtedly the most widely used colorants in our everyday products [6]. Red azo dye TRZ, known as E102 or FD&C Yellow 5, or C.I. 19,140, is a synthetic lemon-yellow colorant used in food, drugs, and cosmetics [7]. Both the FAO/WHO Expert Committee on Food Additives (JECFA) and the European Union Scientific Committee for Food (SCF) determined an acceptable daily intake dose in 1996 [8]. JECFA in 1964 demonstrated TRZ’s identity, purity criteria, and toxicological data and defined an ADI of 0–7.5 mg/kg body weight (bw) [9]. TRZ acceptable daily intake was increased in 2016 from 0–7.5 to 0–10 mg/kg bw based on a NOAL of 984 mg/kg bw due to the lack of compelling evidence of harm at the highest tested doses (≥1000 mg/kg bw/day) in both long-term and reproductive and developmental studies [10].

TRZ is synthesized from coal tar, and it is marketed in the form of water-soluble powder. The levels of TRZ and other synthetic colorants in food product samples can exceed the accepted daily consumption limit in adults and children. Among children, the risk of exceeding ADI is usually much higher than adults, because children are the major consumers of colored food. Thus, exposure to excessive colorants may pose a greater health risk such as hyperactivity [11]. The individual response depends on genetic factors as well as on long-term exposure to low doses [12].

TRZ can be metabolized to an aromatic amine such as sulfanilic acid, which is highly sensitizing to allergies such as urticaria and asthma because TRZ is a nitrous derivative [13,14]. In addition, research has focused on its potential to cause genetic mutations and cancer because it can be converted into an aromatic amine, sulfanilic acid, by the microorganisms in the gut [15]. Sufanilic acid has been shown to cause oxidative stress and cellular damage to human pancreatic cells, for instance [16].

TRZ has been demonstrated to exert histopathological effects on the hepatic and renal tissues of rats, indicated by vacuolation, swelling, necrosis, and pyknosis [17]. Red-azo dye such as TRZ can induce DNA damage in vivo and in vitro [18,19,20,21,22,23]. In one study, the comet assay showed that tartrazine has a genotoxic effect on the white blood cells of rats that were treated [24]. Both TRZ and its metabolites showed genotoxic effects. In addition, TRZ can induce cytotoxicity at high concentrations [24]. The toxic effect of azo dyes has been attributed to the aromatic amines produced from the cleavage of aryl-N=N-aryl [25]. N-hydroxy derivatives, the byproducts of sulfanilic acid, result in neurotoxicity and cause disruptions in redox balance, as evidenced by a significant increase in malondialdehyde (MDA) levels (*p* < 0.05), as well as inhibition of glutathione (GSH) concentration, catalase (CAT), superoxide dismutase (SOD), and glutathione peroxidase (GPx) antioxidant enzyme activities. Additionally, tartrazine-treated rats exhibited elevated levels of acetylcholine (Ach) and gamma-aminobutyric acid (GABA) in the brain, while dopamine (DA) levels were depleted [26].

DNA methyltransferases (DNMTs) and histone deacetylases (HDACs) are groups of enzymes that play a significant role in epigenetics. DNMTs are a family of enzymes that provide a crucial role in genomic stability and integrity. They act on the addition of a methyl group to the 5-position of cytosine residues in DNA. DNA methylation plays a crucial role in regulating gene expression by silencing certain genes by inhibiting their transcription. This can occur through the methylation of promoter regions of genes, preventing the binding of transcriptional factors [27]. There are three active enzymatic members of DNMTs, including DNMT1, DNMT3A, and DNMT3B. Misregulation of them may cause chromosomal instability and carcinogenesis by abnormal DNA methylation. Some studies have shown that DNMT overexpression may lead to the methylation of tumor-suppressor genes, resulting in their silencing and the development of cancer [28]. Hypermethylation of the promoter of DNA repair genes is closely associated with several human tumor types, including colon, breast, and lung cancer [29].

HDACs represent a group of enzymes responsible for the removal of acetyl groups from histones, leading to expressions of several genes. It has been reported that HDACs are responsible for the alteration of acetylation levels, which has been studied in various cancer cells. HDACs could play a significant role in the initiation, progression, and promotion of carcinogenesis [30].

HDAC2, HDAC3, and HDAC8 are all members of the histone deacetylase (HDAC) family of enzymes that play important roles in regulating cell proliferation. They are known to interact with other transcriptional repressors and co-repressors, such as the polycomb group proteins, to silence genes that promote cell proliferation. Studies have shown that *HDAC* genes inhibit cell cycle inhibitors, differentiation, and apoptosis but enhance angiogenesis, invasion, and migration [31,32].

Many food products contain TRZ, which are prepared at different temperatures, such as drinks and juices, cookies, chips, chewing gums, candies, cereals, mustards, dairy products, jellies, ice creams, fillings, liqueurs, powdered juices, soft drinks, yogurts, decoration and coatings, and many other products [33].

This study aimed to evaluate the correlation between TRZ and the expression of *HDAC2*, *HDAC3*, and *HDAC8* as well as *DNMT1*, *DNMT3a*, and *DNMT3b* with the help of qRT-PCR measured in the lungs, kidneys, liver, and spleen of young male and female mice after long-term and short-term exposure, resulting in a detectable enhancing effect of tartrazine on the activity of *DNMT* and *HDAC* genes.

## 2. Materials and Methods

### 2.1. Animals

The selected age of the mice was 6–8 weeks. This corresponds to the human infancy and early sedentary age. The duration of treatment was 30 and 90 days for the mice, which corresponds to approximately 3 and 10 years in human life. Thus, our study targets the life stage and duration at which exposure to tartrazine is greatest in the human population. A total number of 48 male and 48 female NMRI mice (Charles River Laboratories International, Budapest, Hungary) were used in the present study, aged between 6 and 8 weeks, and weighing 30–40 g. The study animals were kept at the Experimental Department (University of Pécs, Pécs, Hungary), where they were housed in standard polycarbonate cages (330 × 160 × 137 mm), bedded with shavings under standard conditions (20–22 °C, humidity 40–60%, 12:12 h light–dark cycle photoperiod) and fed with standard rodent pellet (CRLT/n standard rodent pellet, Sindbad Kft., Gödöllő, Hungary); the water was provided ad libitum. The animal experiment was reviewed and approved by the local authorities (Committee on Research of the University of Pécs, permit number: BA02/2000-12/2018) according to Hungarian animal protection laws in accordance with EU guidelines. In line with ethical guidelines, we reduced the number of animals used and refined the experimental conditions and procedures to minimize harm to animals. The cervical dislocation was performed by a properly trained person in full compliance with the regulations of physical euthanasia.

### 2.2. Treatment of NMRI Mice

NMRI mice were divided into 8 groups of 6 females and 6 males each as described below:

Group I: control group that consumed tap water and rodent chew prepared at room temperature for 30 days.

Group II: control group that consumed tap water and rodent chew prepared at room temperature for 90 days.

Group III: control group that consumed tap water and rodent chew, prepared, and baked after the drying process for 30 min at 160 °C, for the duration of 30 days.

Group IV: control group that consumed tap water and rodent chew, prepared, and baked after the drying process for 30 min at 160 °C, for the duration of 90 days.

Groups V and VI: received special feed consisting of 1×ADI equivalent of human dosage (Human ADI = 7.5 mg/kg/day, which corresponds to 1.845 mg/day of tartrazine, for a mouse with an average body weight of 20 g) [34] of TRZ (Merck-Sigma-Aldrich, Budapest, Hungary), at room temperature for the duration 30 and 90 days, respectively. 

Groups VII and VIII: received special feed consisting of 1×ADI equivalent of human dosage of TRZ prepared similar to the control groups and baked at 160 °C for 30 min for the duration of 30 and 90 days, respectively (Table 1)

### 2.3. Sample Collection and mRNA Isolation

After the respective treatment duration, cervical dislocation was performed. The biopsies were collected from various organs such as lungs, liver, kidneys, and spleen, during necropsy. We isolated total RNA from these tissues using TRIzol reagent (MRTR118-20 NucleotestBio Budapest, Hungary) according to the manufacturer’s protocol. The tissue samples were homogenized using a Polytron homogenizer with TRIzol reagent (1 mL per 50–100 mg of tissue), and 100 µL of chloroform was added to the TRIzol lysate and thoroughly mixed by shaking. After 5 min at room temperature, the samples were centrifuged for 15 min at 12,000× *g* at 4 °C, and the upper aqueous phase containing RNA was collected in a fresh Eppendorf tube. Next, 250 µL of isopropanol was added to the sample, mixed, and kept at room temperature for 10 min before being centrifuged for 10 min at 12,000× *g* at 4 °C. The supernatant was discarded, and the pellet was washed with 70% ethanol and then centrifuged for 5 min at 7500× *g* at 4 °C. The pellet was air-dried, and either RNase-free water or DEPC-treated water was added to the sample. Finally, RNA was quantified using a Nanodrop spectrophotometer.

### 2.4. qRT-PCR

Total RNA was evaluated with Roche Lightcycler 480 (Roche, Basel, Switzerland). Assays were run with the Roche 480 instrument, using the KAPA SYBR FAST One-Step qRT-PCR Master Mix kit (Sigma—Budapest, Hungary). Amplifications were carried out in 20 μL of reaction volume, mixing 5 μL of RNA target (50–100 ng) and 15 μL of the master mix containing forward and reverse primers (10 µL KAPA SYBR FASTqPCR Master Mix, 0.4 µL KAPA RT Mix, 0.4 µL dUTP, 0.4 µL primers (200 nM), 3.8 µL sterile double-distilled water). Reactions were performed with the following thermal profile: 42 °C for 5 min, for the reverse transcription step, a hot-start denaturing step of 95 °C for 3 min, followed by 45 cycles of 95 °C for 10 s (denaturation), 60 °C for 20 s (annealing/extension). The fluorogenic signal emitted was read during the annealing–extension step and was analyzed by software. Immediately after amplification, a melting curve protocol was produced by increasing each cycle by 0.5 °C, starting from the set-point temperature (55.0 °C), for 80 cycles, each one at 10 s. The primary sequences of the housekeeping gene used as an internal control, hypoxanthine–guanine phosphoribosyltransferase (*HPRT1*), and the genes of interest, *DNMT1*, *DNMT3A*, *DNMT3B*, *HDAC2*, *HDAC3*, and *HDAC8*, are shown in Table 2. Primers were designed with Primer Express™ Software (Applied Biosystems, Budapest, Hungary) and were synthesized by Integrated DNA Technologies (Bio-Sciences, Budapest, Hungary). The results were analyzed by the relative quantification (2^−∆∆CT^) method.

### 2.5. Statistical Analysis

To evaluate our results, we performed an ANOVA test, Levene’s F test, and then a post hoc analysis (Scheffe and LSD test). The Kolmogorov–Smirnov test was applied to determine the distribution and standard deviation. Statistical analysis was performed using IBM SPSS statistics for Windows, Version 26.0 (Armonk, NY, USA). Significance was established at *p* < 0.05.

## 3. Results

### 3.1. Expression of DNMT Genes

**Figure 1:**
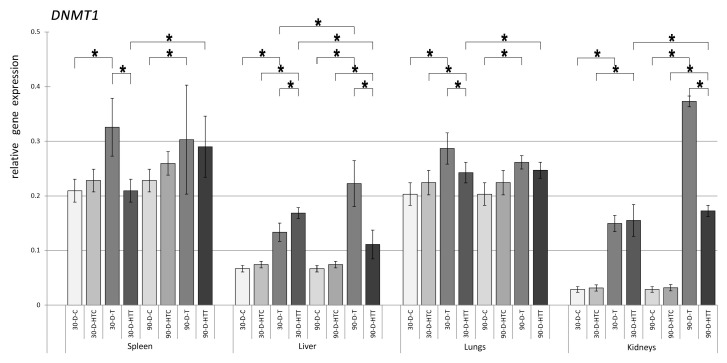
Relative gene expression level of DNMT1 in the spleen, liver, lungs, and kidneys of the animals, *p* < 0.05, after 30- and 90-day consumption of tartrazine prepared at room temperature and treated with high heat (30-D-C: 30-day control, 30-D-HTC: 30-day heat-treated control, 30-D-T: 30-day consumption of TRZ, 30-D-HT: 30-day consumption of heat-treated TRZ, 90-D-C: 90-day control, 90-D-HTC: 90-day heat-treated control, 90-D-T: 90-day consumption of TRZ, 90-D-HT: 90-day consumption of heat-treated TRZ). (* = *p* < 0.05).

The gene expression of epigenetics-related enzymes was measured in the samples taken from the NMRI mice’s spleen, liver, lungs, and kidneys. When *DNMT1* expression (Figure 1) was measured in the spleen, liver, lungs, and kidneys, the 30-day and 90-day room temperature TRZ-treated groups had significantly elevated levels compared to the control groups. When TRZ-containing feed was prepared at high temperatures, significant activation of *DNMT1* was observed in the liver, lungs, and kidneys from the 30-day treatment and the liver and kidneys from the 90-day treatment. Lengthening the treatment from 30 to 90 days significantly activated *DNMT1* expression in the liver and kidneys from the room-temperature TRZ and the spleen, liver, and lungs from the high-temperature TRZ groups. When we examined how the temperature of the chew preparation influenced *DNMT1* expression, the preparation at room temperature had higher gene expression in the spleen, lungs, and kidneys of the 30-day and the liver and kidneys of the 90-day groups.

**Figure 2:**
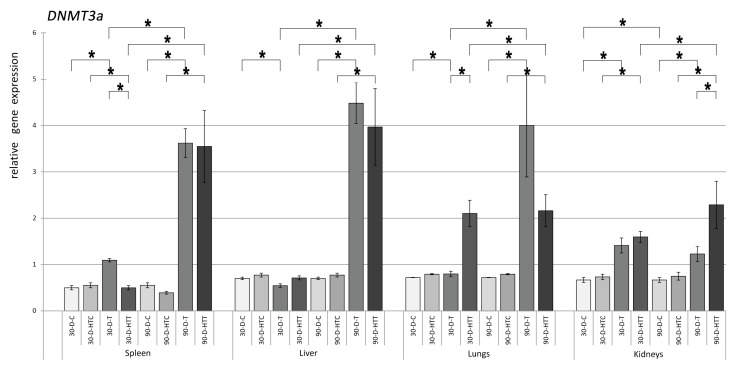
Relative gene expression level of DNMT3a in the spleen, liver, lungs, and kidneys of the animals, *p* < 0.05, after 30- and 90-day consumption of tartrazine prepared at room temperature and treated with high heat (30-D-C: 30-day control, 30-D-HTC: 30-day heat-treated control, 30-D-T: 30-day consumption of TRZ, 30-D-HT: 30-day consumption of heat-treated TRZ, 90-D-C: 90-day control, 90-D-HTC: 90-day heat-treated control, 90-D-T: 90-day consumption of TRZ, 90-D-HT: 90-day consumption of heat-treated TRZ). (* = *p* < 0.05).

For the expression of the *DNMT3a* gene (Figure 2), 30- and 90-day room-temperature TRZ-treated samples from the spleen, liver, lungs, and kidneys indicated a significant increase compared to the corresponding control groups. In high-temperature TRZ groups, the spleen and kidneys of 30-day and all tissue samples of the 90-day groups expressed higher *DNMT3a*. Significantly higher *DNMT3a* expressions were observed in the 90-day treatment group than in the 30-day one for spleen, liver, lungs, and kidneys in both unheated and heated TRZ conditions. When the preparation temperatures of the mice chews were analyzed, only lung samples in the 90-day group had significant differences.

**Figure 3:**
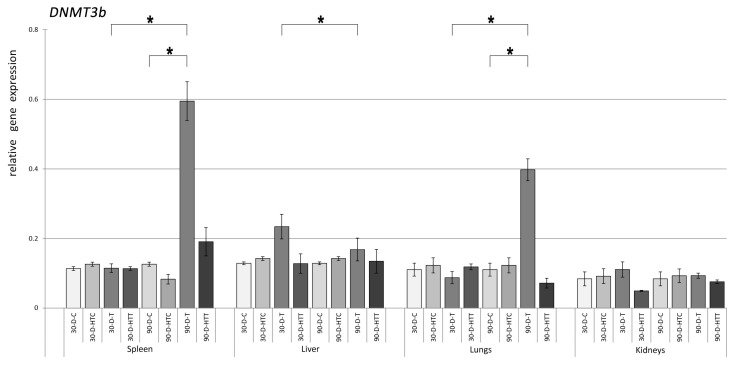
Relative gene expression level of DNMT3b in the spleen, liver, lungs, and kidneys of the animals, *p* < 0.05, after 30- and 90-day consumption of tartrazine prepared at room temperature and treated with high heat (30-D-C: 30-day control, 30-D-HTC: 30-day heat-treated control, 30-D-T: 30-day consumption of TRZ, 30-D-HT: 30-day consumption of heat-treated TRZ, 90-D-C: 90-day control, 90-D-HTC: 90-day heat-treated control, 90-D-T: 90-day consumption of TRZ, 90-D-HT: 90-day consumption of heat-treated TRZ). (* = *p* < 0.05).

*DNMT3b* gene expression was not statistically different (Figure 3) among all 30- and 90-day room-temperature TRZ treatment groups and their respective control groups, except for the 90 days of tartrazine treatment in the spleen sample. However, during the high-temperature TRZ treatment, the liver sample from 30 days and the spleen and liver from 90 days had increased *DNMT3b* expression compared to their controls. Significant changes were observed in the spleen and liver for 30 days and the spleen and lungs for 90 days when TRZ was prepared at high temperatures.

### 3.2. Expression of HDAC Genes

**Figure 4:**
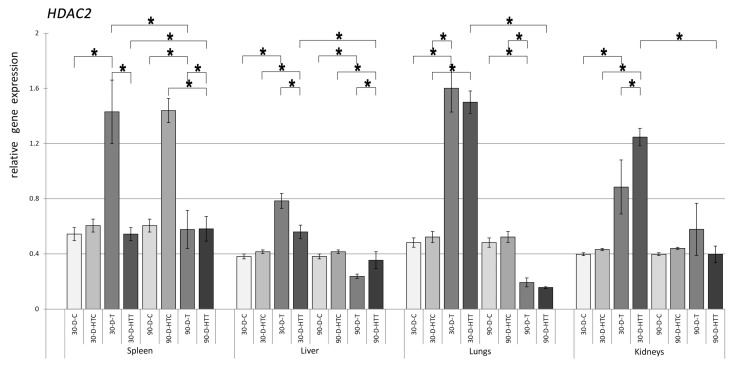
Relative gene expression level of HDAC2 in the spleen, liver, lungs, and kidneys of the animals, *p* < 0.05, after 30- and 90-day consumption of tartrazine prepared at room temperature and treated with high heat (30-D-C: 30-day control, 30-D-HTC: 30-day heat-treated control, 30-D-T: 30-day consumption of TRZ, 30-D-HT: 30-day consumption of heat-treated TRZ, 90-D-C: 90-day control, 90-D-HTC: 90-day heat-treated control, 90-D-T: 90-day consumption of TRZ, 90-D-HT: 90-day consumption of heat-treated TRZ). (* = *p* < 0.05).

Elevated levels of *HDAC2* expression were observed (Figure 4) in the spleen, liver, lungs, and kidneys of the 30- and 90-day room temperature TRZ-treated groups compared to the control groups. When the TRZ was prepared at a high temperature, significant activation of *HDAC2* was found in the liver, lungs, and kidneys of the 30-day group and in the spleen, liver, lungs, and kidneys of the 90-day group. The increase in treatment duration from 30 to 90 days resulted in significant activation of *HDAC2* expression in the spleen of the room-temperature TRZ and the spleen and liver of the high-temperature TRZ group. The gene expression of *HDAC2* was significantly different in the spleen, lungs, and kidneys of the 30-day group and the spleen of the 90-day group when the different chew preparation temperatures were compared.

**Figure 5:**
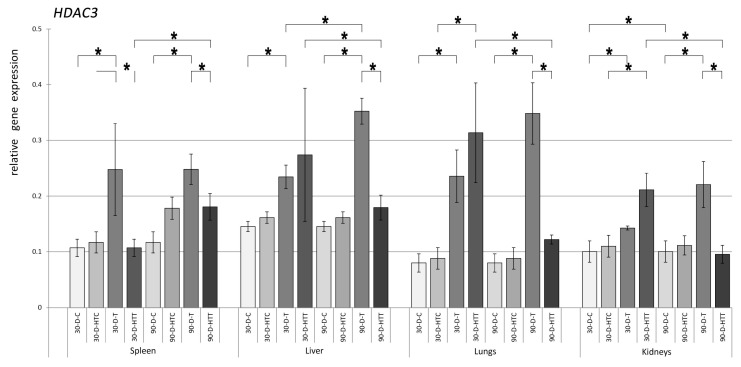
Relative gene expression level of HDAC3 in the spleen, liver, lungs, and kidneys of the animals, *p* < 0.05, after 30- and 90-day consumption of tartrazine prepared at room temperature and treated with high heat (30-D-C: 30-day control, 30-D-HTC: 30-day heat-treated control, 30-D-T: 30-day consumption of TRZ, 30-D-HT: 30-day consumption of heat-treated TRZ, 90-D-C: 90-day control, 90-D-HTC: 90-day heat-treated control, 90-D-T: 90-day consumption of TRZ, 90-D-HT: 90-day consumption of heat-treated TRZ). (* = *p* < 0.05).

**Figure 6:**
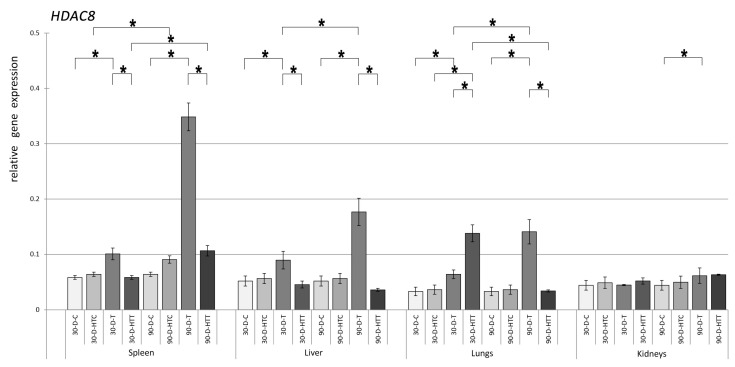
Relative gene expression level of HDAC8 in the spleen, liver, lungs, and kidneys of the animals, *p* < 0.05, after 30- and 90-day consumption of tartrazine prepared at room temperature and treated with high heat (30-D-C: 30-day control, 30-D-HTC: 30-day heat-treated control, 30-D-T: 30-day consumption of TRZ, 30-D-HT: 30-day consumption of heat-treated TRZ, 90-D-C: 90-day control, 90-D-HTC: 90-day heat-treated control, 90-D-T: 90-day consumption of TRZ, 90-D-HT: 90-day consumption of heat-treated TRZ). (* = *p* < 0.05).

The expression pattern of the *HDAC3* gene was very similar to that of *HDAC2*, but there were some differences (Figure 5). It was also found that, in general, *HDAC3* expression was lower than that of *HDAC2*. Nevertheless, there was a clearly significant increase in *HDAC3* expression caused by TRZ. The greatest difference was observed in the liver and lungs, where TRZ diets prepared at room temperature after 90 days of administration resulted in a relatively higher significant increase in *HDAC3* expression compared to *HDAC2*. The expression levels of the *HDAC8* gene were significantly increased (Figure 6) in the spleen, liver, and lungs of both 30- and 90-day room-temperature TRZ-treated groups compared to the control groups that did not receive TRZ treatment. However, in the kidneys, only the 90-day TRZ treatment group showed a significant increase in expression compared to the control. When TRZ was prepared at a high temperature, none of the samples tested caused significant changes in *HDAC8* expression. When the treatment duration was increased from 30 to 90 days, there was a significant rise in *HDAC8* expression in the spleen, liver, and kidneys of room-temperature TRZ and the spleen of the high-temperature TRZ group. In addition, the gene expression of *HDAC8* was found to be substantially different in the spleen, liver, and lungs of both the 30- and 90-day groups, depending on the temperature at which the chew preparation was made.

In summary, the results show that tartrazine affected the activity of all the genes tested in the *HDAC* and *DNMT* families. In addition, all organs were affected. However, the strongest response varied depending on which time point, which organ, and which gene.

## 4. Discussion

Our study examined the impact of the acceptable daily intake of tartrazine, a food azo dye, on a set of genes of DNA epigenetic modifiers, *DNMT*s, and *HDAC*s.

The widespread use of dye in the food industry has raised significant concerns regarding its excessive utilization in food products. Although the literature is available on the toxicity of food azo dyes at both high and low doses, little information is available on the ADI of these dyes. A range of azo dye products display genotoxic properties only when they are reduced by enteral bacteria such as *Enterococcus faecalis*. This mechanism appears to be linked to the formation of superoxide and oxygen radicals [35]. Azo compounds comprise an aromatic ring that is connected via an azo bond to a second naphthalene or benzene ring [12]. After oral consumption, azo compounds are reduced by azo reductases present in intestinal bacteria and the liver’s cytosolic and microsomal enzyme fraction. This process results in the formation of aromatic amines, which have been detected in the urine of experimental animals and paint industry workers following the administration of food azo dyes [36].

It has been reported that gastrointestinal microflora can metabolize tartrazine and produce aromatic amine sulfanilic acid [15]. Consequently, there is a possibility that these aromatic amines may produce reactive oxygen species, including hydrogen peroxide, superoxide anion, and hydroxyl radical, during their metabolism. This could potentially lead to oxidative stress [37]. Based on oxidative stress, TRZ induced dose-related DNA damage in rat colons, which may lead to the formation of tumor cells [38]. Peroxynitrite, a potent RNA formed by the reaction of nitric oxide and ROS, may also play a role in DNA damage [39].

To study the long-term effects, Himri and colleagues (2011) found that ADI doses of TRZ and its consequent metabolic byproducts, namely, sulfanilic acid, have the ability to produce reactive oxygen species (ROS), which leads to oxidative stress and potentially impacts the hepatic and renal structures as well as their respective biochemical profiles [40]. Administration of tartrazine led to a significant increase in plasma aspartate transaminase (AST), alanine transaminase (ALT), and alkaline phosphatase (AlP) levels compared to the control group (*p* < 0.05). Additionally, there was a significant reduction in the total antioxidant capacity observed following tartrazine treatment [41]. The increased levels of malondialdehyde (MDA), which is a product of lipid peroxidation, and nitric oxide (NO) in the rats treated with tartrazine strongly suggest the occurrence of oxidative stress [42]. Related to our study, reactive oxygen species (ROS) can trigger the overexpression of *DNMT*s, which can result in DNA hypermethylation [43]. In the present study, tartrazine significantly increased *DNMT1*, *DNMT3a*, *HDAC2*, and *HDAC3* in both liver and kidneys along with *HDAC8* in the kidney tissues, but the level of *DNMT3b* did not change noticeably in the kidney.

There is limited research on the specific molecular pathways in the lungs that may be affected by tartrazine. However, some studies have suggested that tartrazine may induce oxidative stress and inflammation [44]; however, it may contribute to lung damage and exacerbation of respiratory conditions such as asthma. Ram Prasad’s research group discovered that elevated levels of prostaglandin E2 and cyclooxygenase-2 (Cox-2) are associated with increased *DNMT* activity in the epithelial cells [45]. In current research, tartrazine has elevated the relative expression of *DNMT*s and *HDAC*s, which suggests that tartrazine may have a negative effect on lung health by inducing oxidative stress, inflammation, and upregulation of pro-inflammatory cytokines. However, further research is needed to fully understand the mechanisms underlying these effects.

Recently, there have been several studies published regarding the effect of epigenetic mechanisms such as *HDAC*s and *DMNT*s overexpression in the brain. One study conducted by Valera et al. suggested that modulating the *DNMT* and *HDAC* enzymes could potentially contribute to the manifestation of anti-manic effects. This was concluded because effectively regulating these enzymes has been observed to reverse the effects of Ouabain (OUA), an inhibitor of Na+/K+ATPase. OUA is known to extend the depolarized state of neurons and to induce hyperactivity [46]. Another study by Dash et al. found that the occurrence of traumatic brain injury (TBI) triggers a multifaceted cascade of neurochemical and signaling transformations that give rise to a range of pathological consequences. These include heightened neuronal activity, an excessive release of glutamate, inflammation, increased permeability of the blood–brain barrier (BBB), cerebral edema, modified gene expression, and impaired neuronal function. The administration of valproate shortly after a traumatic brain injury has the potential to provide neuroprotection. This medication can exert its protective effects by inhibiting the activity of *GSK-3* and *HDAC*s [47]. Wang et al. demonstrated that a brief treatment with the class I HDAC inhibitors MS-275 or romidepsin improved social and cognitive impairments in transgenic mice carrying the 16p11.2 deletion (16p11del/+). These findings strongly indicate that inhibiting HDACs can serve as an immensely effective therapeutic approach for addressing behavioral deficits in 16p11del/+ mice [48]. The mentioned research has concluded that *HDAC* and *DNMT* genes can alleviate conditions such as mania, hyperactivity, bipolar disorders, neurological disorders, and behavior deficits, which can be reversed with medications that act as HDAC inhibitors, such as valproate. In the current study, we witnessed an overexpression of these genes in several organs due to the exposure of the experimental animals to tartrazine; meanwhile, tartrazine and its metabolites can pass the blood–brain barrier and induce neurotoxicity. Although we gained data from different organs other than the brain, we hypothesize that tartrazine may play a role in behavior and neurological disorders from consuming a food product that meets the current regulations regarding ADI. We believe that further investigations of everyday doses of tartrazine are needed to study the long-term impact on brain function.

## 5. Conclusions

This is the first study to investigate the effects of oral administration of tartrazine on *DNMT* and *HDAC* genes over a long treatment period while keeping the recommended dose of intake. The present study shows that tartrazine can cause a change in the gene expressions of *DNMT1*, *DNMT3a*, *DNMT3b*, *HDAC2*, *HDAC3*, and *HDAC8* over a long period even at a dose at the ADI level in different organs. Based on the data, compared with the literature, we hypothesize that tartrazine consumption may contribute to cell proliferation and may even increase the likelihood of carcinogenesis. Furthermore, the observed effect in various organs highlights the possibility that the genes under investigation may also be activated in the brain, with proven consequences for brain function. 

In view of these findings, it is of great importance to investigate the direct effect of tartrazine on brain function, and replacing the artificial mono-azo dyes with natural food dyes in the food industry, where it is necessary to color food and beverages, should be considered. 

## Figures and Tables

**Table 1:** Treatment and sample preparation for the eight groups. The experimental NMRI mice received a daily amount of laboratory rodent chew mixed with an equivalent human dose of TRZ for the duration of the experiment.

Groups	Duration of Consumption	Temperature of Sample Preparation
Group 1 Control	30 days	Room temperature
Group 2 Control	90 days	Room temperature
Group 3 Control	30 days	160 °C
Group 4 Control	90 days	160 °C
Group 5 + TRZ	30 days	Room temperature
Group 6 + TRZ	90 days	Room temperature
Group 7 + TRZ	30 days	160 °C
Group 8 + TRZ	90 days	160 °C

**Table 2:** Sequences of primers used for relative gene expression level measure with qRT-PCR.

Gene name	Forward Primer	Reverse Primer
DNA methyltransferase 1 (*DNMT1*)	AAGAATGGTGTTGTCTACCGAC	CATCCAGGTTGCTCCCCTTG
DNA methyltransferase 3A (*DNMT3a*)	GAGGGAACTGAGACCCCAC	CTGGAAGGTGAGTCTTGGCA
DNA methyltransferase 3B (*DNMT3b*)	AGCGGGTATGAGGAGTGCAT	GGGAGCATCCTTCGTGTCTG
Histone deacetylase 2 (*HDAC2*)	GGAGGAGGCTACACAATCCG	TCTGGAGTGTTCTGGTTTGTCA
Histone deacetylase 3 (*HDAC3*)	GCCAAGACCGTGGCGTATT	GTCCAGCTCCATAGTGGAAGT
Histone deacetylase 8 (*HDAC8*)	ACTATTGCCGGAGATCCAATGT	CCTCCTAAAATCAGAGTTGCCAG
Hypoxanthine phosphoribo-syltransferase 1 (*HPRT1*)	TCAGTCAACGGGGGACATAAA	GGGGCTGTACTGCTTAACCAG

## Data Availability

Data will be made available on request.
